# Early lens aging is accelerated in subjects with a high risk of ischemic heart disease: an epidemiologic study

**DOI:** 10.1186/1471-2415-6-16

**Published:** 2006-04-18

**Authors:** Line Kessel, Torben Jørgensen, Charlotte Glümer, Michael Larsen

**Affiliations:** 1 Department of Ophthalmology, Herlev Hospital, University of Copenhagen, Denmark; 2 Research Centre for Prevention and Health, Copenhagen County, Glostrup University Hospital, Denmark; 3 Steno Diabetes Center, Gentofte, Denmark

## Abstract

**Background:**

Ischemic heart disease (IHD) is one of the most important causes of mortality and morbidity in the Western world. There is a relationship between aging of the lens of the human eye and cardiovascular disease. The present study was conducted to examine if the risk of ischemic heart disease could be estimated by fluorophotometric assessment of lens aging.

**Methods:**

A total of 421 subjects were included. Risk of IHD was estimated from non-ocular data using the Precard ^® ^software. Lens aging was quantified by lens fluorometry.

**Results:**

The risk of IHD was strongly related to lens fluorophore accumulation (p = 0.001). The relationship between IHD and lens aging was attributable to tobacco smoking and dysglycemia.

**Conclusion:**

The risk of ischemic heart disease related to smoking and diabetes mellitus can be estimated using the aging of the lens of the eye as a biomarker for generalized tissue-damage.

## Background

Ischemic heart disease (IHD) is the most common cause of death in the Western world [1]. Major risk factors are diabetes mellitus, smoking, and dyslipidemia [2-4]. An association between cataract and cardiovascular disease has been demonstrated previously [5,6]. Cataract is an opacification of lens of the human eye interfering with visual function. Senile cataract is a common disease associated with oxidative stress, [7,8] smoking, [9,6,3,11] and diabetes mellitus [6,12].

The association between aging of the lens and ischemic heart disease may well be a causative one. Both lens proteins, plasma proteins, and the collagen of the vascular walls are subject to denaturation by a spontaneous reaction between aminogroups and reducing sugars, [13] glycotoxins from tobacco smoke, [14] or lipid peroxidation products [15]. The spontaneous reaction leads to formation of advanced glycation end products (AGEs). Advanced glycation end products have been shown to increase atherogenicity of LDL-particles [16] as well as playing a role in stiffening of arterial vessel walls [17] both of which are important features in the pathogenesis of IHD. In the lens of the human eye, AGEs lead to accumulation of yellow, fluorescent compounds [18,19] thus causing the intrinsic fluorescence [20].

Targeting premorbid cardiovascular disease prevention in individuals at high risk depends upon the availability of biomarkers of long-term morbidity and mortality risk. At present, detection of subjects at high risk for IHD is based on risk assessment from a combination of informations from blood tests, lifestyle, and genetic predisposition [21]. Substituting these with a single measurement or biomarker would be advantageous. The search for a non-invasive biomarker for cardiovascular disease is ongoing [22-24]. Cataract is a marker of the risk of and/or presence of cardiovascular disease, but the grading of cataract and its subclinical precursors is generally subjective and crude. We hypothesized that assessment of age-related changes in the lens could be used as a biomarker for IHD. The intrinsic fluorescence of the lens is an alternative objective parameter of lens aging that can be measured fast, non-invasively, and without pupil dilation. Another objective measurement of lens ageing, Scheimpflug photography, exists. However, it requires medical pupil dilation which makes it a less attractive instrument in the present context. In the present study we compared lens fluorometry with an established method of prediction of ischemic heart disease risk in healthy individuals.

## Methods

### Study population

Subjects were enrolled from a prospective epidemiologic study on the prevalence of ischemic heart disease (IHD) and effect of non-pharmalogical intervention on IHD risk [25]. Details of subject recruitment and characteristics have been described thoroughly previously [25]. In brief, participants in the Inter99 Study were drawn on a random basis from the Danish Civil Registration System which comprises all subjects residing permanently in Denmark. Participants were recruited in 7 birth cohorts (years of birth 1939/1940, 1944/1945, 1949/1950, 1954/1955, 1959/1960, 1964/1965, and 1969/1970).

The present study was part of The Inter99 Eye Study which consists of invited subjects from the Inter99 Study. The Inter99 Eye Study population was invited in two main groups: 1) an age- and sex-stratified control group polled to match the background population and 2) high risk subjects, i.e. subjects with high risk of ischemic heart disease or subjects with diabetes mellitus or impaired glucose tolerance. The present study includes only subjects from the control group (potentially eligible, n = 502). The study population thus represents the entire spectrum of IHD risk and glucose tolerance. Thirty-six subjects were excluded as they did not have lens fluorescence measured due to technical difficulties. Furthermore, 2 subjects were excluded due to previous corneal grafting (keratoplasty), and 10 eyes of 6 subjects were excluded due to cortical lens opacities exceeding 2.5 on the LOCS III grading scale as such opacities may interfere with the fluorescence measurements because they reach into the central 2 mm of the optical axis where fluorescence measurements take place [26]. Only subjects who had an eye examination within 6 months of the general health assessment were included, this criteria lead to exclusion of 39 subjects. Thus, a total of 421 subjects were included in the present study.

The present study population matched the background population which was a random sample of the Danish suburban population as assessed on the basis of a number of important characteristics such as age and IHD risk (table 1).

**Table 1:** Comparison of the study population to the background population.

	Study population	Inter99 background population	p-value
Sex (males/females)	181 (43.0)/240 (57.0)	3121 (49.0)/3242 (51.0)	0.02*
Age (years)	46.6 (8.1)	46.0 (8.0)	0.14
IHD risk (%)	4.5 (1.0–65.8)	4.8 (0.4–77.0)	0.53§
Fasting p-glucose (mmol/l)	5.4 (3.0–19.7)	5.4 (2.0–23.9)	0.23§
120-min p-glucose (mmol/l)	5.9 (2.5–25.9)	5.9 (1.4–30.6)	0.90§

Ischemic heart disease (IHD) risk was calculated as the risk of having an IHD event projected to occur between the age of 60 and 70 years using the Precard ^® ^software. [21] Data are shown in mean (SD) for age and median (range) for IHD risk, fasting p-glucose, and 120-min post OGTT p-glucose, or actual numbers (%) for sex. P-values were calculated using the Students t-test when data were normally distributed or the non-parametric equivalent, the Mann-Whitney U-test (§) when data were not normally distributed. * A χ^2^-test was used.

The study protocol was reviewed and approved by the local medical ethics committee of Copenhagen County. Written informed consent was obtained from all participants after the nature and possible consequences of the study had been carefully explained.

### Procedures

All subjects underwent a general ophthalmic examination including determination of visual acuity and assessment of fundus status. Subjects were asked about present or past history of ophthalmic disorders, surgery, or ocular medication, family history of cataract and glaucoma.

Lens fluorescence was measured on the undilated eye using a non-invasive ocular fluorophotometer, the Fluorotron (OcuMetrics, Mountain View, CA, USA). Excitation was from 430–490 nm and detection from 530 to 630 nm. The Fluorotron was fitted with an anterior segment adaptor that concentrates the 149 measurement steps in intraocular intervals of 0.125 mm in the anterior segment of the eye. Lens fluorescence was measured along the optical axis of the eye and the measurements were corrected for loss of light due to absorption as described previously. [27] Six scans, three from each eye, were recorded and the mean of these 6 scans was used in the analyses. All fluorescence values were calibrated to an external solution of fluorescein and are reported in ng fluorescein equivalents per ml [ng f-eq/ml].

Presence and severity of lens opacities was determined from retroilluminated lens photographs focused on the anterior lens surface taken after dilation of the pupil using one drop of 0.5% tropicamide and one drop of 10% phenylephrine hydrochloride on 35 mm Kodak Ektachrome 64 colour slides (Kodak Inc, Rochester, NY, USA) using a Canon 60UVi camera (Canon Inc, Japan). The degree of cortical lens opacities was graded by one of the authors (LK) according to the LOCS III scale. [28] Cortical lens opacities exceeding 2.5 on the LOCS III scale lead to exclusion of that eye. Nuclear lens aging was assessed by lens autofluorometry using the Fluorotron. Lens fluorescence measurements have previously been shown to correlate well to the LOCS III nuclear grading [29].

Furthermore, all subjects underwent a thorough general health examination and questionnaire concerning past and present medical history, and use of medication. A physical examination was performed including determination of height, weight, waist circumference, and ECG. Venous blood samples were drawn for measurement of plasma cholesterol, HDL-cholesterol, triglycerides, and HbA1c. An oral glucose tolerance test was used and evaluated according to WHO 1999 criteria [30].

Ischemic heart disease (IHD) risk was calculated using the Precard ^® ^software. The Precard software calculates the risk (in percentage) of having an ischemic heart event based on age, sex, cholesterol (total cholesterol and HDL-cholesterol), systolic blood pressure, smoking, body mass index, diabetes mellitus, family history of cardiovascular disease and previous heart disease. [21] As the risk of ischemic heart disease increases dramatically with age, an age-standardized measure was produced by projecting the ischemic heart event to occur between the ages of 60 and 70 years.

Smoking was assessed as number of pack years ever smoked. One pack year equals 20 cigarettes per day for one year.

### Statistical procedures

All statistical procedures were performed using the SAS software (version 8.2, SAS Institute, Cary, NC, USA). The level of statistical significance was set at 0.05. Lens fluorescence was transformed to logarithmic values to obtain a normal distribution with a constant variance, all fluorescence values reported have been backtransformed to the geometrical mean with 95% confidence intervals. Relations between variables were calculated using linear or multiple regression analysis.

## Results

Lens fluorescence demonstrated a strong relationship to age (r = 0.70, p < 0.0001). The results presented below for association between lens fluorescence and other parameters are reported after correction for the effect of age by multiple regression analysis.

Clinical characteristics of the study population (n = 421) is shown in table 2.

**Table 2:** Clinical characteristics of the study population.

Number of subjects	421
Males/females	181/240
NGT/IGT/DM*	321/43/41
Smoker (never, occasionally, ex, present)	159/41/105/116
Previous AMI (yes/no)	9/412
Family history of CVD (yes/no)	34/387
Age (years)	46.6 (8.1)
Lens fluorescence (ng f-eq/ml)	421.0 (212.0–832.4)
HbA1c (%)	5.8 (4.3–14.4)
Fasting p-glucose (mmol/l)	5.4 (3.0–19.7)
2-hour OGTT p-glucose (mmol/l)	5.9 (2.5–25.9)
Cholesterol	5.6 (1.0)
HDL-cholesterol	1.4 (0.4)
BMI (kg/m^2^)	30.8 (24.9–46.3)
Waist circumference (cm)	86.2 (14.3)
Pack years	0.5 (0–90.0)
Systolic blood pressure (mmHg)	129.3 (15.9)
Diastolic blood pressure (mmHg)	81.8 (9.9)

NGT denotes normal glucose tolerance, IGT impaired glucose tolerance, DM diabetes mellitus (both known and newly diagnosed), AMI acute myocardial infarction. CVD cardiovascular disease. BMI body mass index. One pack year equals 20 cigarettes per day for one year and is calculated as tobacco ever smoked. Never smokers were defined as subjects who had never smoked tobacco and ever smokers were defined as all those who had smoked and includes both present and ex-smokers. Body mass index (BMI) was calculated as weight divided by the square height (in meters). Values are given in mean (SD) except for pack years, HbA1c, BMI, fasting p-glucose, and 2-hour OGTT p-glucose that are given in median (range) as they are not normally distributed. Lens fluorescence is given as mean (95% confidence interval) after a backtransformation from logarithmic values. *The oral glucose tolerance test was not performed in 16 subjects.

### Lens fluorescence in relation to ischemic heart disease risk

A significant positive association was found between lens fluorescence (LF, log10 values) and ischemic heart disease risk (IHD risk, in %) (p = 0.001, adjusted R^2^= 51.4%), eq. 1:

LF = 0.0126·Age + 0.0024·IHD risk + 2.0214     (eq. 1)

**Figure 1:**
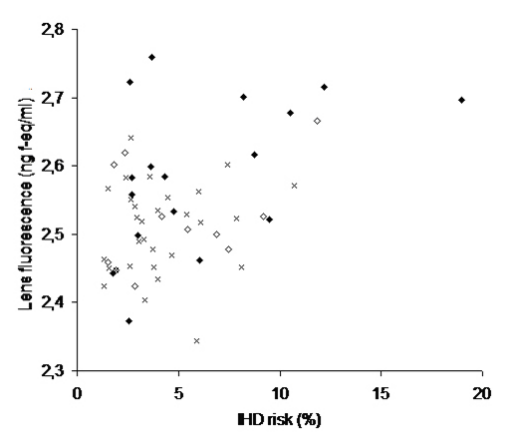
Lens fluorescence in relation to ischemic heart disease risk. Analysis of the entire study population (n = 421) demonstrated that the risk of ischemic heart disease increased with increasing lens fluorescence (p = 0.001), the association being statistically attributable to tobacco smoking and increasing levels of glycemia. Because of the strong increase in lens fluorescence with age, only subjects who were 40 years of age at time of examination is shown in the graph (n = 73). Lens fluorescence is shown in logarithmic values. Ischemic heart disease risk was estimated using the Precard ^® ^software. Subjects who had smoked 10 pack years or more are dipicted as filled diamonds (◆), subjects who had smoked less than 10 pack years are dipicted as open diamonds (◇), and subjects who had never smoked are shown as crosses (x).

Risk of IHD was calculated as a subject's risk of having an IHD event projected to occur between the age of 60 and 70 years. Figure 1 shows the relationship between lens fluorescence and IHD risk.

Risk of having an ischemic heart disease (IHD) event, projected to occur between the age of 60 and 70 years, was calculated using the Precard^® ^software. Since lens fluorescence was significantly increased in subjects with high IHD risk profile we wanted to examine whether this was due to one or more of the specific variable(s) that were associated to IHD risk.

Lens fluorescence was significantly related to age (p < 0.0001) and number of pack years ever smoked (p < 0.0001), and it was significantly increased in subjects with diabetes mellitus compared to normoglycemic subjects (p < 0.0001). No relation was found for lens fluorescence and sex (p = 0.37), or lens fluorescence and total cholesterol (p = 0.13), HDL-cholesterol (p = 0.44), systolic blood pressure (p = 0.99), or body mass index (p = 0.17). Lens fluorescence was not increased in subjects who had previously had a myocardial infarction (n = 11, p = 0.28) or in subjects with a family history of cardiovascular disease (n = 34, p = 0.99).

In order to quantify the effect of dysglycemia on lens fluorescence, three different glucose variables were tested: HbA1c, fasting p-glucose and 120-min post oral glucose tolerance test p-glucose. All three variables showed a significant positive association to lens fluorescence (p-values < 0.0001, Pearsons correlation coefficients ranged from 0.30 to 0.34) as well as to IHD risk assessed using the Precard ^® ^software (p-values < 0.0001, Pearsons correlation coefficients ranged from 0.40 to 0.49). Equation 2 comprises the single IHD risk factors that showed a significant relationship to lens fluorescence (p < 0.0001 for all three explanatory variables, adjusted R^2 ^= 54.0%). Fasting p-glucose was chosen to represent dysglycemia.

LF = 0.01191·Age + 0.01637·Fasting glucose + 0.00127·Pack years + 1.96338     (eq. 2)

Thus, association between lens fluorescence and the Precard ^® ^IHD risk estimate (eq. 1) was as strong as the relationship between lens fluorescence and the known single risk factors for IHD, hyperglycemia and tobacco smoking (eq. 2), adjusted R^2 ^0.51 and 0.54, respectively.

## Discussion

We have demonstrated a distinct association between ocular lens fluorescence and the estimated risk of ischemic heart disease (IHD) calculated on the basis of a number of non-ocular health parametres. The relationship was attributable to the effects of glucose metabolism and tobacco smoking on both the aging process of the lens and IHD risk. Glucose metabolism and tobacco smoking are well-known risk factors for both lens aging [27] and cardiovascular disease [31,2]. Glucose and substances in tobacco smoke, glycotoxins, induce irreversible changes on proteins through formation of advanced glycation end products leading to impaired collagen function such as stiffening of arterial walls [32,33,13]. In the lens, these protein changes are marked features of the aging process and they can be quantified non-invasively and in vivo by lens autofluorometry. The effect on the rest of the body excerted by these factors can, however, not be assessed in vivo unless invasive procedures are used.

Ischemic heart disease is a complex disease with atherosclerosis being an important aetiologic factor. Atherosclerosis is a multifactorial process [34]. Advanced glycation end products may play a role in atherosclerosis through several pathways such as crosslinking of collagen in the vascular wall thereby reducing elasticity and increasing vascular tension, [17,35] by increasing atherogenicity of LDL-particles, [16] and by quenching nitric oxide and thereby increasing vascular tension [36]. The results from the present study indicate that assessment of lens aging can be a way of estimating the extent of tissue-damage related to glucose and glycotoxins in tobacco smoke.

Tissue-damage by non-enzymatic glycation is just one of many features leading to IHD. Important risk factors such as cholesterol levels showed no association to lens fluorescence. A risk estimate of IHD covering the entire IHD risk spectrum can thus not be obtained by lens autofluorometry. Nevertheless, lens autofluorometry may provide a useful window to parts of the body that are usually concealed. Given the high blood glucose levels in patients with diabetes mellitus, assessment of tissue-damage related to advanced glycation end products may be most valuable in patients with diabetes mellitus.

Previous studies have focused on identifying non-invasive biomarkers for cardiovascular disease in order to assess the extent of tissue-damage [22,24,23]. Morphological changes of the arterioles on the retina of the eye have been demonstrated to be a marker for the long-term effect of hypertension and to be related to the risk of cerebrovascular disease [22]. The results of the present study suggests that lens fluorescence can be used as an indicator of tissue-damage caused by advanced glycation end products and that lens fluorescence can be used to give an estimate of the risk of IHD related to the effect of advanced glycation end products. Prospective, follow-up studies are, however, needed to demonstrate whether lens fluorometry yields better overall risk estimates than the conventional method and whether risk profiling of healthy subjects can be refined by utilizing fluorometry data.

## Conclusion

In conclusion, the present study suggests that a common risk estimate of ischemic heart disease related to tobacco smoking and diabetes mellitus can be obtained by lens autofluorometry. Presumably, a combination of ocular parameters such as lens fluorometry and retinal photographs will provide useful information about the degree of tissue-damage excerted on the body by a number of risk factors for cardiovascular disease and thus summarize the extent of body damage.

## Abbreviations

AGE advanced glycation end products. IHD ischemic heart disease

## Declaration of competing interests

The author(s) declare that they have no competing interests.

## Authors' contributions

LK made substantial contributions to the conception and design of the Inter99 Eye Study, was involved in patient examination, data management, and performed all statistical analyses. Furthermore, LK drafted the manuscript. TJ and CG made substantial contributions to the conception and design of the Inter99 Study. TJ was the main intellectual resource on data concerning ischemic heart disease and CG was the main intellectual resource on data concerning diabetes mellitus. ML made substantial contributions to the conception and design of the Inter99 Eye study and to the analysis and interpretation of data. All authors have contributed intellectually to the manuscript and have given final approval of the version to be published.

## Pre-publication history

The pre-publication history for this paper can be accessed here:



## References

[B1] Sans S, Kesteloot H, Kromhout D (1997). The burden of cardiovascular diseases mortality in Europe. Task Force of the European Society of Cardiology on Cardiovascular Mortality and Morbidity Statistics in Europe. Eur Heart J.

[B2] Bhargava A (2003). A longitudinal analysis of the risk factors for diabetes and coronary heart disease in the Framingham Offspring Study. Popul Health Metr.

[B3] Hiller R, Sperduto RD, Podgor MJ, Wilson PW, Ferris FLIII, Colton T, D'Agostino RB, Roseman MJ, Stockman ME, Milton RC (1997). Cigarette smoking and the risk of development of lens opacities. The Framingham studies. Arch Ophthalmol.

[B4] Beks PJ, Mackaay AJ, de Neeling JN, de Vries H, Bouter LM, Heine RJ (1995). Peripheral arterial disease in relation to glycaemic level in an elderly Caucasian population: the Hoorn study. Diabetologia.

[B5] Hu FB, Hankinson SE, Stampfer MJ, Manson JE, Colditz GA, Speizer FE, Hennekens CH, Willett WC (2001). Prospective study of cataract extraction and risk of coronary heart disease in women. Am J Epidemiol.

[B6] Delcourt C, Cristol JP, Tessier F, Leger CL, Michel F, PApoz L, group TPOLA (2000). Risk factors for cortical, nuclear, and posterior subcapsular cataracts: the POLA study.. Am J Epidemiol.

[B7] Spector A (2000). Review: Oxidative stress and disease. J Ocul Pharmacol Ther.

[B8] Chylack LTJ (1984). Mechanisms of senile cataract formation. Ophthalmology.

[B9] Arnarsson A, Jonasson F, Sasaki H, Ono M, Jonsson V, Kojima M, Katoh N, Sasaki K (2002). Risk factors for nuclear lens opacification: the Reykjavik Eye Study. Dev Ophthalmol.

[B10] Hirvelä H, Luukinen H, Laatikainen L (1995). Prevalence and risk factors of lens opacities in the elderly in Finland. A population-based study. Ophthalmology.

[B11] West S, Munoz B, Emmett EA, Taylor HR (1989). Cigarette smoking and risk of nuclear cataracts. Arch Ophthalmol.

[B12] Leske MC, Chylack LTJ, Wu SY (1991). The Lens Opacities Case-Control Study. Risk factors for cataract. Arch Ophthalmol.

[B13] Ulrich P, Cerami A (2001). Protein glycation, diabetes, and aging. Recent Prog Horm Res.

[B14] Nicholl ID, Bucala R (1998). Advanced glycation endproducts and cigarette smoking. Cell Mol Biol (Noisy -le-grand).

[B15] Fu MX, Requena JR, Jenkins AJ, Lyons TJ, Baynes JW, Thorpe SR (1996). The advanced glycation end product, Nepsilon-(carboxymethyl)lysine, is a product of both lipid peroxidation and glycoxidation reactions. J Biol Chem.

[B16] Makita Z, Yanagisawa K, Kuwajima S, Bucala R, Vlassara H, Koike T (1996). The role of advanced glycosylation end-products in the pathogenesis of atherosclerosis. Nephrol Dial Transplant.

[B17] Wolffenbuttel BH, Boulanger CM, Crijns FR, Huijberts MS, Poitevin P, Swennen GN, Vasan S, Egan JJ, Ulrich P, Cerami A, Levy BI (1998). Breakers of advanced glycation end products restore large artery properties in experimental diabetes. Proc Natl Acad Sci U S A.

[B18] Das BK, Sun TX, Akhtar NJ, Chylack LTJ, Liang JJ (1998). Fluorescence and immunochemical studies of advanced glycation-related lens pigments. Invest Ophthalmol Vis Sci.

[B19] Lyons TJ, Silvestri G, Dunn JA, Dyer DG, Baynes JW (1991). Role of glycation in modification of lens crystallins in diabetic and nondiabetic senile cataracts. Diabetes.

[B20] Abiko T, Abiko A, Ishiko S, Takeda M, Horiuchi S, Yoshida A (1999). Relationship between autofluorescence and advanced glycation end products in diabetic lenses. Exp Eye Res.

[B21] Thomsen TF, Davidsen M, Jørgensen T, Ibsen H, Jensen G, Borch-Johnsen K (2001). A new method for CHD prediction and prevention based on regional risk scores and randomized clinical trials; PRECARD and the Copenhagen Risk Score. J Cardiovasc Risk.

[B22] Wong TY, Klein R, Klein BE, Tielsch JM, Hubbard L, Nieto FJ (2001). Retinal microvascular abnormalities and their relationship with hypertension, cardiovascular disease, and mortality. Surv Ophthalmol.

[B23] Schwartz RS, Bayes-Genis A, Lesser JR, Sangiorgi M, Henry TD, Conover CA (2003). Detecting vulnerable plaque using peripheral blood: inflammatory and cellular markers. J Interv Cardiol.

[B24] Pahor M, Elam MB, Garrison RJ, Kritchevsky SB, Applegate WB (1999). Emerging noninvasive biochemical measures to predict cardiovascular risk. Arch Intern Med.

[B25] Jørgensen T, Borch-Johnsen K, Thomsen TF, Ibsen H, Glümer C, Pisinger C (2003). A randomised non-pharmacological intervention study for prevention of ischemic heart disease. Baseline results. Inter99 (1). Eur J Cardiovasc Prevention Rehab.

[B26] Siik S, Airaksinen PJ, Tuulonen A, Nieminen H (1993). Autofluorescence in cataractous human lens and its relationship to light scatter. Acta Ophthalmol (Copenh).

[B27] Kessel L, Hougaard JL, Sander B, Kyvik KO, Sørensen TI, Larsen M (2002). Lens ageing as an indicator of tissue damage associated with smoking and non-enzymatic glycation - a twin study. Diabetologia.

[B28] Chylack LTJ, Wolfe JK, Singer DM, Leske MC, Bullimore MA, Bailey IL, Friend J, McCarthy D, Wu SY (1993). The Lens Opacities Classification System III. The Longitudinal Study of Cataract Study Group. Arch Ophthalmol.

[B29] Siik S, Chylack LTJ, Friend J, Wolfe J, Teikari J, Nieminen H, Airaksinen PJ (1999). Lens autofluorescence and light scatter in relation to the lens opacities classification system, LOCS III. Acta Ophthalmol Scand.

[B30] (1999). Definition, diagnosis and classification of diabetes mellitus and its complications. Report of a WHO Consultation. Part 1: Diagnosis and classification of diabetes mellitus. WHO/NCD/NCS/99 2.

[B31] Alberti KG (1996). The clinical implications of impaired glucose tolerance. Diabet Med.

[B32] Monnier VM, Kohn RR, Cerami A (1984). Accelerated age-related browning of human collagen in diabetes mellitus. Proc Natl Acad Sci U S A.

[B33] Cerami C, Founds H, Nicholl I, Mitsuhashi T, Giordano D, Vanpatten S, Lee A, Al Abed Y, Vlassara H, Bucala R, Cerami A (1997). Tobacco smoke is a source of toxic reactive glycation products. Proc Natl Acad Sci U S A.

[B34] Lusis AJ (2000). Atherosclerosis. Nature.

[B35] Sims TJ, Rasmussen LM, Oxlund H, Bailey AJ (1996). The role of glycation cross-links in diabetic vascular stiffening. Diabetologia.

[B36] Bucala R, Tracey KJ, Cerami A (1991). Advanced glycosylation products quench nitric oxide and mediate defective endothelium-dependent vasodilatation in experimental diabetes. J Clin Invest.

